# Does Amifostine Reduce Metabolic Rate? Effect of the Drug on Gas Exchange and Acute Ventilatory Hypoxic Response in Humans

**DOI:** 10.3390/ph8020186

**Published:** 2015-04-16

**Authors:** Jaideep J. Pandit, Caroline Allen, Evelyn Little, Federico Formenti, Adrian L. Harris, Peter A. Robbins

**Affiliations:** 1 Nuffield Department of Anaesthetics, Oxford University Hospitals, Oxford OX3 9DU, UK; 2 Department of Physiology, Anatomy and Genetics, Parks Road, Oxford OX1 3PT, UK; 3 Molecular Oncology, Weatherall Institute of Molecular Medicine, University of Oxford, OX3 9DU, UK

**Keywords:** hypoxia, hypercapnia, ventilation, ventilatory control, chemoreflexes, metabolism, gas exchange, hypoxia-inducible factor

## Abstract

Amifostine is added to chemoradiation regimens in the treatment of many cancers on the basis that, by reducing the metabolic rate, it protects normal cells from toxic effects of therapy. We tested this hypothesis by measuring the metabolic rate (by gas exchange) over 255 min in 6 healthy subjects, at two doses (500 mg and 1000 mg) of amifostine infused over 15 min at the start of the protocol. We also assessed the ventilatory response to six 1 min exposures to isocapnic hypoxia mid-protocol. There was no change in metabolic rate with amifostine as measured by oxygen uptake (*p* = 0.113). However in carbon dioxide output and respiratory quotient, we detected a small decline over time in control and drug protocols, consistent with a gradual change from carbohydrate to fat metabolism over the course of the relatively long study protocol. A novel result was that amifostine (1000 mg) increased the mean ± SD acute hypoxic ventilatory response from 12.4 ± 5.1 L/min to 20.3 ± 11.9 L/min (*p* = 0.045). In conclusion, any cellular protective effects of amifostine are unlikely due to metabolic effects. The stimulatory effect on hypoxic ventilatory responses may be due to increased levels of hypoxia inducible factor, either peripherally in the carotid body, or centrally in the brain.

## 1. Introduction

The drug amifostine (whose active metabolite is 2-((aminopropyl)amino)ethanethiol) is added to the chemoradiation regimens in the treatment of several cancers to protect normal healthy cells from the toxic effects of treatment and to reduce side effects such as xerostomia. Amifostine increases rates of response in several types of tumour, including tumours of the uterine cervix, oesophagus, rectum, small-cell lung cancers, and locally advanced carcinomas of the head and neck [1]. The precise mode of action has not been fully elucidated. One possibility is that its conversion to a disulfide enables subsequent scavenging of free radicals, thus protecting the normal cell against DNA and cell membrane damage by chemo- radiotherapy [2]. Notably, metabolic conversion of amifostine to WR33278 results in reduced oxygen consumption of healthy tissue [3]. This observation was based on the finding that venous haemoglobin saturation and P_O2_ increased with amifostine administration in patients. However, this observation was subsequently questioned by Koukourakis *et al.* (2004) [4], who reported a biphasic response. Although haemoglobin saturation and P_O2_ rose slightly at 30 min after administration, at 60 min there was a more profound fall in these measures. No previous study has measured the effect of amifostine on metabolic rate (e.g., by gas exchange) in humans.

Furthermore amifostine may, directly or indirectly, upregulate the expression of a variety of proteins, including HIF-1α, involved in the process of DNA repair and apoptosis inhibition [4,5]. It has been reported that HIF-1α is involved in carotid body transduction of hypoxia, such that increased levels (or increased stability of HIF-1α) augments the ventilatory hypoxic response [6,7]. Therefore, if amifostine increases HIF-1α levels in the carotid body, it might also have the effect of augmenting the ventilatory hypoxic response.

In relation to the drug amifostine, these two lines of investigation are contradictory. If some of the cancer therapeutic literature is correct and amifostine reduces whole-body metabolic rate, then we might expect it to reduce the magnitude of hypoxic ventilatory response. This is for the reason that, in conditions of increased metabolic rate such as exercise, hypoxic ventilatory response is consistently increased [8,9]. However, if the line of enquiry relating to HIF-1α and the carotid body is correct, we would expect the hypoxic ventilatory response to be increased. It is important in the field of cancer therapeutics that the actions of drugs like amifostine is known. It is also suggested that manipulation of metabolic rate may be an important aspect of outcomes in anaesthesia and intensive care [10]. We therefore wished to test two hypotheses. First, we wished to establish if (as suggested by the cancer therapeutic literature) metabolic rate is in fact reduced by amifostine. This would be an important and novel finding as we are not aware of any drug or therapy that reduces basal metabolic rate in humans. Second, we wished to assess by direct measurement whether hypoxic ventilatory response is increased or reduced by amifostine.

## 2. Experimental Section

After Oxford Research Ethics Committee approval (06/Q1606/117) and written informed consent, 6 healthy subjects (3 females) were studied; their mean ± SD age was 24.8 ± 8.7 yrs, weight 73.0 ± 7.1 kg, height 174 ± 5 cm. Metabolic gas exchange was assessed over a 255 min period of continuous air breathing, against a background infusion of: (a) 500 mg amifostine; (b) 1000 mg amifostine; or (c) control saline, in random order (sealed envelope selection, in advance), on different days. Female subjects were studied in the same phase of their menstrual cycle. Amifostine was administered over 15 min and at doses in accordance with clinical protocols [4,11]. The assessment of acute hypoxic response was conducted mid-way during this protocol (time = 100 min) over a ~20 min period. The end-tidal gas profile was: an initial 10 min period of air breathing (end tidal partial pressure carbon dioxide, PET_CO2_ 40 Torr, end tidal partial pressure oxygen PET_O2_ 100 Torr), followed by six 1 min steps of isocapnic hypoxia (PET_O2_ 50 Torr) alternating with 1 min recovery (PET_CO2_ 40 Torr, PET_O2_ 100 Torr).

Infusion of amifostine was conducted into a forearm vein at the rate of 10 mL/h. Amifostine (Sigma-Aldrich, Poole, UK) was dissolved in saline to achieve a concentration of 20 or 40 mg/mL for the 500 mg and 1000 mg doses, respectively. Because amifostine has been reported to cause nausea and vomiting, subjects were pre-treated with 8 mg of intravenous ondansetron and 10 mg of intravenous metoclopramide (which were also administered in control protocols).

The control of end-tidal gases in all protocols was achieved using a computer-controlled gas-mixing system, described in detail elsewhere [12,13]. Briefly, time profiles for inspiratory P_CO2_ and P_O2_ that were likely to produce the desired sequences in PET_CO2_ and PET_O2_ were predicted and imposed during the experiment. Actual PET_CO2_ and PET_O2_ values as measured by mass spectrometer were fed back into the computer breath-by-breath, compared with the desired values, and adjusted breath-by-breath using a system of gas cylinders controlled by fast-responding valves to force the PET_CO2_ and PET_O2_ towards the desired values. Subjects sat comfortably, breathing through a mouthpiece with their nose occluded. Respiratory volumes were recorded using a bi-directional turbine-measuring device.

Heart rate and blood oxygen saturation were measured continuously and blood pressure was measured at regular intervals in all protocols. Blood samples were taken on every hour and analysed for erythropoietin (EPO) and vascular endothelial growth factor (VEGF) concentrations.

Data were averaged over 30 s intervals and analyzed using our EXHALE program to determine O_2_ consumption ($\dot{V}$O_2_) and CO_2_ production ($\dot{V}$CO_2_) at the mouth, as well as minute ventilation. The method has been described elsewhere [14,15], but briefly: the records of the CO_2_ and O_2_ composition at the mouth were time-aligned with that of respiratory flow, using the mass spectrometer delay time. Then, the flow values were adjusted for changes in viscosity due to changes in gas composition. Third, for each half breath, the respiratory flow profile was calibrated for volume. Fourth, for each gas, the difference between the amount breathed in and the amount breathed out was calculated, using an assumed expired temperature. This assumed expired temperature was then adjusted until it resulted in the net nitrogen exchange over the experimental period being equal to zero.

Respiratory quotient (RQ) was then calculated as $\dot{V}$CO_2_/$\dot{V}$O_2_. The mean of each 10 min period of mouth piece breathing was calculated to assess $\dot{V}$O_2_, $\dot{V}$CO_2_ and RQ. These values were available breath-by-breath but where results showed linear trends, any change in metabolic variables was calculated by subtracting the start-point average (the 10 min average of the first period of mouth piece breathing at t = 15) with the end-point average (the 10 min average of the last period of mouth piece breathing at t = 240). The 30 s periods before the onset of hypoxia and the highest response during the 1 min period of hypoxia were used to calculate the acute hypoxic response, with each protocol provided multiple hypoxic periods.

The study was powered to detect a difference in the main outcome measure (oxygen consumption) of 50 mL/min. Previous work [9] indicates that the standard deviation of this change in human subjects is ~25 mL/min; Lehr’s formula thus estimates a sample size of 4 for 80% power at the 5% level, so 6 subjects achieve at least this power [16].

Results were assessed using factorial analysis of variance (ANOVA), with factors being “subject”, “drug”, and (for metabolic data) “time” [17]. *Post-hoc* differences were assessed using t-tests. Significance was assumed when *p* < 0.05.

## 3. Results

**Figure 1:**
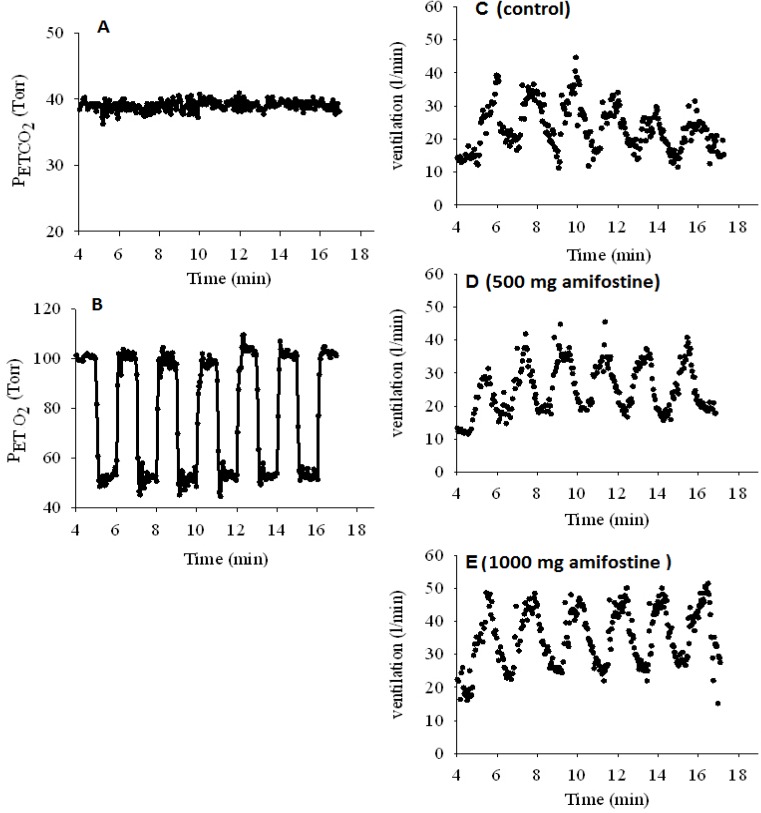
Panels A and B: Gas input protocol for one example subject for the ~20 min period of measurement of hypoxic ventilatory response. Panel A, PET_CO2_; Panel B, PET_O2_ profile. Panels C–E: Ventilatory responses for one example subject for the ~20 min period of measurement of hypoxic ventilatory response. Panel C, control; Panel D, with 500 mg amifostine; Panel E, with 1000 mg amifostine.

Figure 1 (A–B) shows the gas input profile and ventilatory response for one example subject. Gas control was good with sharp steps into and out of hypoxia while PET_CO2_ was held suitably constant. Figure 1 (C–E) shows the resulting ventilatory responses. It appeared that amifostine, in increasing doses, increased the response to hypoxia and perhaps, at the highest dose, increased basal ventilation.

**Figure 2:**
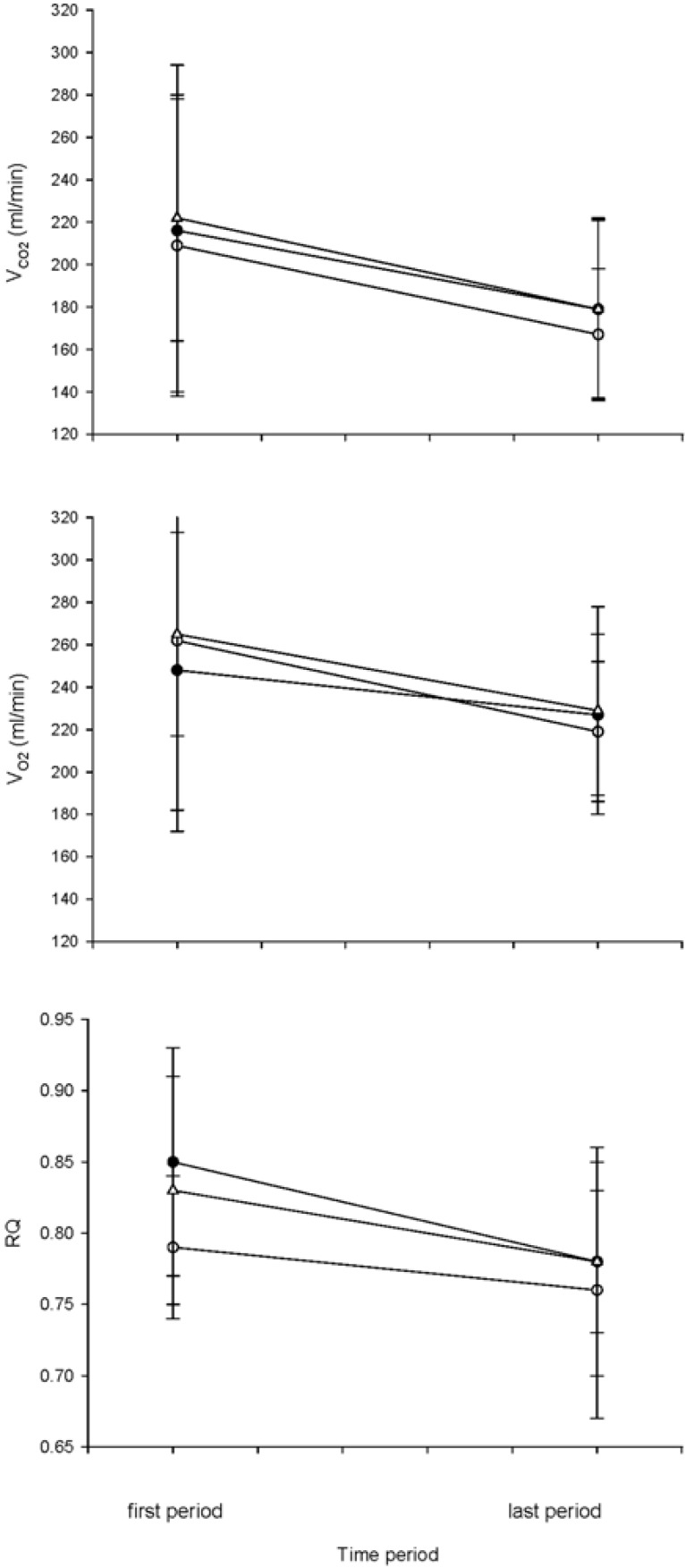
Mean ± SD for all subjects, of $\dot{V}$CO_2_ (Panel A), $\dot{V}$O_2_ (Panel B) and RQ over time for control (●), 500 mg amifostine (○) and 1000 mg amifostine (△) protocols. The “first period” represents the mean of values over the first 10 min of data collection (time = 15–25 min) and the “last period” to the last 10 min of data collection (time = 240–255 min).

Figure 2 shows the metabolic gas exchange data for all subjects combined. There appears to be a decline over time in $\dot{V}$CO_2_, but less so in $\dot{V}$O_2_ such that RQ declines over time. However, there does not seem to be a pronounced drug effect. This was confirmed by data analysis. ANOVA for $\dot{V}$CO_2_ indicated significant effects over “time” (*p* = 0.038), but not of “drug” (*p* = 0.532) or of the interactive term (“drug” * “time”, *p* = 0.935). However, the downward trend in $\dot{V}$O_2_ over time was not significant (*p* = 0.113), nor was there a significant “drug” effect (*p* = 0.765). Thus, the corresponding downward trend in RQ over time was significant (ANOVA; *p* = 0.001). Again, this reduction was not dependent on variation in “drug” (*p* = 0.677) or of the interactive term (*p* = 0.321).

Table 1 shows the data for each individual and for the group as a whole. Amifostine did not have an effects on baseline ventilation (the “drug” effect in ANOVA NS, *p* = 0.402). However, the drug increased hypoxic ventilatory response (“drug” effect in ANOVA *p* = 0.016); *post-hoc* t-tests located this effect to the comparison of high-dose *vs.* control (*p* = 0.045) and to high-dose *vs*. low-dose (*p* = 0.027), but not to the comparison of low-dose amifostine *vs.* control (*p* = 0.491).

**Table 1:** Effects of amifostine on basal minute ventilation and the acute hypoxic response (absolute values). Control, 500 mg and 1000 mg amifostine shown in columns, in order.

Subject no.	Baseline ventilation; mean (SD) (L/min)				Hypoxic ventilatory response; mean (SD) (L/min)		
	Control	500 mg	1000 mg		control	500 mg	1000 mg
1096	12.8 (1.9)	9.1 (0.6)	9.4 (1.4)		10.3 (2.8)	8.0 (2.8)	9.8 (2.6)
1529	10.1 (0.7)	7.0 (0.5)	6.8 (1.5)		7.4 (0.5)	4.9 (1.0)	11.3 (1.7)
1599	12.2 (1.6)	10.6 (0.5)	9.7 (0.8)		5.9 (0.8)	4.9 (3.4)	8.5 (2.0)
1638	12.0 (0.9)	12.1 (3.4)	20.4 (0.9)		17.6 (3.8)	23.2 (3.0)	36.7 (4.0)
1646	14.4 (1.4)	12.3 (0.6)	20.6 (2.7)		16.1 (5.2)	24.4 (3.3)	26.2 (1.8)
1649	12.3 (1.8)	19.1 (1.9)	18.8 (3.1)		16.8 (2.0)	16.9 (1.9)	29.0 (5.0)
Mean	12.3	11.7	14.3		12.4	13.7	20.3
SD	1.4	4.1	6.3		5.1	9.0	11.9

There was no significant alteration in VEGF plasma levels (mean 74.7 pg/mL) with ‘drug’ (ANOVA, *p* = 0.756) or “time” (ANOVA, *p* = 0.406). EPO concentrations (mean 7.1 pg/mL) also did not vary with “drug” (ANOVA, *p* = 0.430) or “time” (ANOVA, *p* = 0.920).

Cardiovascular responses were assessed because amifostine can cause hypotension but there were no changes in heart rate or blood pressure with amifostine (data not shown) and all subjects were cardiovascularly stable throughout the protocols.

## 4. Discussion

We report the novel finding that high doses of amifostine markedly increased hypoxic ventilatory response. Furthermore, contrary to reports from the cancer therapeutic literature, we did not find significant influence of the drug on whole-body metabolic rate.

In contrast to earlier suggestions that amifostine might reduce metabolic rate based on isolated tissue samples in microcalorimetry [18] or based on only indirect assessment venous blood gas levels [3], we found no reduction in $\dot{V}$CO_2_ or $\dot{V}$O_2_ (and thus, RQ) with amifostine in whole-body study. Instead, we report an incidental small decline in time in $\dot{V}$CO_2_ and RQ (19% and 7%, respectively) across all our protocols, including the control protocol. We ascribe this to the effect of starvation. The experimental period was rather long (255 min) and the RQ declined from a value of ~0.8 indicating carbohydrate metabolism to one of ~0.7 closer to fat metabolism (Figure 2). This is a well-known phenomenon in short-term starvation during both rest and exercise [14,19].

Our results in relation to hypoxic response are consistent with the notion that amifostine is thought to increase levels of (or to stabilise) hypoxia inducible factor (HIF), a transcription factor which binds to hypoxia response elements in the promoters of target genes. Yet, we did not find any increase in plasma VEGF or EPO, which might have been expected with HIF activation. However, some studies report that VEGF can be suppressed by hypoxia and our hypoxic tests might have counteracted any tendency for VEGF plasma levels to increase as a result of amifostine infusion [20]. If amifostine stimulates HIF we might have expected to observe a rise in EPO. In retrospect this expectation may have been misplaced as amifostine in fact has a limited effect on plasma EPO levels or haemoglobin [21], *i.e.*, its effects are probably confined to tissues rather than detectable systemically. Note that not all previous studies have reported a stimulation of amifostine on HIF: Dedieu *et al.* failed to find an effect [22], albeit their findings were confined to tumour cell lines.

*In vivo* experiments with breast tumour cell lines show amifostine administration results in HIF1α induction and in rats results in increased HIF1α accumulation in normal tissue [4]. Though the exact cellular mechanism of oxygen sensing and signalling in the carotid body cells is not known, there is an increasing body of evidence implicating the involvement of HIF. Isolated carotid bodies of mice that are heterozygous for a knockout allele at the *Hif1a* locus show little increase in carotid sinus nerve activity in response to acute hypoxia. *Hif1a^+/−^* mice show impaired physiological responses to chronic hypoxia [23,24].

Based on this evidence, an upregulatory effect of amifostine on the HIF pathway in the carotid body could cause an increased ventilatory response to hypoxia, but the precise mechanism of how this is achieved within the carotid body is not known. In fact, carotid-chemodenervated rats can later develop ventilatory responses to sustained hypoxia, associated with upregulation of tyrosine hydroxylase in brainstem catecholaminergic neurones [25], suggesting that O_2_-sensitive mechanisms can (under certain circumstances) develop in the brain. In cultured PC12 cells, regulation of tyrosine hyodoxylase transcription has been shown to require HIF activation [26] and Pascual *et al.* (2001) reported that HIF1-α is selectively expressed in medullary regions during sustained hypoxia in rats, especially in glial cells [27]. Amifostine or its metabolites do not cross the blood brain barrier, but it has been advocated as a neuroprotective agent because it can influence the biochemistry of the cerebral vascular endothelium [28]. It is possible therefore, that the results we describe may be mediated by (indirect) central nervous system effects of amifostine (or HIF) rather than peripheral carotid body mechanisms, and this possibility warrants specific study.

Our results refute the hypothesis that amifostine’s protective effect is via a reduction in basal metabolism. Our finding of increased hypoxic ventilatory response is consistent with its previously established upregulation of HIF. However, these findings raise a new dilemma that warrants explanation: upregulation of HIF is not consistent with a cellular protective effect since HIF activation is generally thought to be associated with promoting tumorigenic activity [29]. Our results therefore require fundamental re-analysis of the mode of action of amifostine.
